# The Distinct Electrophysiological Mechanisms in the Cortico-Striatal Circuit of LID Rats

**DOI:** 10.3390/biology15131074

**Published:** 2026-07-04

**Authors:** Tingting He, Hongyu Wang, Haoqi Ni, Yuting Sun, Xiang Gao, Fan Zhou, Jianmin Zhang, Kedi Xu

**Affiliations:** 1 Key Laboratory of Biomedical Engineering of Education Ministry, Department of Biomedical Engineering, Zhejiang University, Hangzhou 310027, China; 12115030@zju.edu.cn (T.H.); hongyu_w@zju.edu.cn (H.W.); nihq@zju.edu.cn (H.N.); sunyt@zju.edu.cn (Y.S.); 13858000009@139.com (F.Z.); 2 Zhejiang Key Laboratory of Intelligent Sensing Technology and Advanced Medical Instrument, Department of Biomedical Engineering, Zhejiang University, Hangzhou 310027, China; 3 Binjiang Institute of Zhejiang University, Hangzhou 311000, China; gao_x@zju.edu.cn; 4 Department of Neurosurgery, The Second Affiliated Hospital, Zhejiang University School of Medicine, Hangzhou 311100, China; zjm135@vip.sina.com; 5 The State Key Laboratory of Brain-Machine Intelligence, Zhejiang University, Hangzhou 311100, China

**Keywords:** levodopa-induced dyskinesia, single-neuron spikes, local field potentials, the dorsolateral striatum, the primary motor cortex, phase-amplitude coupling, delta, gamma

## Abstract

Levodopa-induced dyskinesia (LID) is a severe motor complication associated with long-term levodopa (L-DOPA) treatment for Parkinson’s disease (PD). Its underlying mechanisms remain unclear, and candidate biomarkers lack consistency. To investigate cortico-striatal network alterations associated with LID, we simultaneously recorded single-neuron spikes and local field potentials (LFPs) from the dorsolateral striatum (DLS) and the primary motor cortex (M1) in LID rats. Our results showed that in the DLS, the LID group had a greater number of putative fast-spiking interneurons (FSIs) with lower firing rates, and fewer putative medium spiny neurons (MSNs) with higher firing rates. In M1, pyramidal neurons were fewer but fired faster, while interneurons were more numerous with no change in firing rate. Although gamma power increased and delta power decreased in both regions in LID rats, delta-gamma phase-amplitude coupling (PAC) was present in the DLS but absent in M1. Furthermore, cross-regional PAC analysis revealed significantly stronger coupling between the low-frequency phase of M1 and the high-frequency amplitude of the DLS than in the opposite direction, indicating an asymmetric pattern of cortico-striatal coupling in LID. These findings demonstrate region-specific alterations in neuronal activity and oscillatory coupling associated with LID and suggest that asymmetric cortico-striatal PAC may serve as a promising electrophysiological marker for characterizing abnormal network dynamics underlying dyskinesia.

## Simple Summary

Parkinson’s disease is a neurodegenerative disorder characterized primarily by motor dysfunction. The main treatment, levodopa, works well at first but often causes severe, uncontrollable movements after long-term use, a condition called levodopa-induced dyskinesia (LID). The underlying neural mechanisms of LID are not yet fully understood. In this study, we used a rat model to investigate how neuronal activity is altered following chronic levodopa treatment. We recorded electrical signals from two brain areas involved in the motor cortex and the dorsolateral striatum in rats with this condition. Our results showed abnormal patterns of neural activity in both regions, including altered neuronal firing rates and disrupted interregional coordination. Importantly, we discovered that the coupling strength between M1’s low-frequency phase and DLS’s high-frequency amplitude exceeds that of the reverse pairing (DLS phase–M1 amplitude) in the LID model, which may reflect an electrophysiological feature associated with dyskinesia. Our findings contribute to a better understanding of the neural mechanisms underlying LID and suggest that this specific communication pathway may represent a promising therapeutic target for future interventions.

## 1. Introduction

Parkinson’s disease (PD) is a progressive neurodegenerative disorder characterized by the degeneration of dopaminergic neurons in the substantia nigra pars compacta. This pathology results in dopamine depletion in the striatum, and the subsequent emergence of motor symptoms including bradykinesia, rigidity, and tremor [1,2]. Although levodopa (L-DOPA) remains the most effective treatment for alleviating these motor symptoms, prolonged treatment frequently results in levodopa-induced dyskinesia (LID). This complication affects more than half of PD patients and severely impairs their quality of life [3,4]. LID is characterized by involuntary, excessive movements that greatly restrict the therapeutic benefits of L-DOPA [5]. Despite considerable advances in PD research, the neural mechanisms underlying LID remain incompletely understood.

The pathophysiology of LID is closely associated with abnormal activity within the cortico-basal ganglia network, particularly an imbalance between excitatory and inhibitory signalling induced by dopamine depletion and pulsatile dopaminergic stimulation [6]. Within this network, the dorsolateral striatum (DLS) acts as a critical integrative hub receiving cortical and dopaminergic inputs [7], while the primary motor cortex (M1) functions as a major output region translating neural activity into motor behaviour [8]. Increasing evidence suggests that disrupted cortico-striatal coordination contributes to the development of LID. However, the electrophysiological mechanisms by which chronic L-DOPA exposure reshapes electrophysiological interactions within this circuit remain incompletely understood.

Neural oscillations play an important role in the temporal coordination of neuronal activity within and across brain regions [9,10]. In both PD and LID, pathological changes in oscillatory activity have been widely reported [11,12]. For example, exaggerated beta-band oscillations are strongly associated with the parkinsonian state [13,14,15], whereas enhanced gamma-band activity has been linked to dyskinesia [16]. In addition to spectral power changes, cross-frequency coupling provides additional information about the temporal organization of neural dynamics [17]. In particular, phase-amplitude coupling (PAC) enables low-frequency oscillations to regulate the timing and excitability of high-frequency activity [18]. PAC not only reflects the dynamic properties of local neural circuits but is also considered an electrophysiological marker of coordinated activity across brain regions. Previous studies have reported abnormal beta–gamma or theta–gamma coupling in PD and LID [9,19,20]. However, the role of delta-mediated PAC in cortico-striatal coordination during LID has not been systematically investigated.

Importantly, neural activity is organized across multiple spatial and temporal scales, ranging from single-neuron firing to population-level oscillations. Single-unit recordings characterize neuronal firing patterns and cell type-specific activity [21,22], whereas local field potentials (LFPs) reflect the synchronized activity of local neuronal populations and synaptic inputs [23]. Together with phase–amplitude coupling (PAC), these complementary electrophysiological measures provide insight into neuronal activity at different levels of circuit organization [24,25,26,27]. Nevertheless, the interactions among these electrophysiological features, particularly across brain regions, have not been systematically investigated.

In this study, we simultaneously recorded single-unit activity and local field potentials (LFPs) from the dorsolateral striatum (DLS) and primary motor cortex (M1) in a rat model of LID. By integrating spike classification, spectral analysis, and intra- and inter-regional phase–amplitude coupling (PAC) analyses, we aimed to characterize multi-scale electrophysiological alterations within the cortico-striatal circuit and to identify electrophysiological features associated with LID that may serve as potential biomarkers for future therapeutic strategies.

## 2. Materials and Methods

### 2.1. Animals

Male Sprague–Dawley rats (280–320 g) were obtained from the Nanhu Brain–Computer Interface Institute. Animals were housed under controlled conditions (22 ± 2 °C) on a 12 h light/dark cycle with ad libitum access to food and water. All experimental procedures were approved by the Animal Care and Use Committee of Zhejiang University and conducted in accordance with institutional guidelines.

### 2.2. 6-OHDA Lesion and Model Validation

Rats were randomly assigned to three groups: sham (n = 15), PD (n = 15), and LID (n = 15). To establish unilateral PD models, 30 rats were anesthetized with sodium pentobarbital (Sigma, St. Louis, MI, USA, 30 mg/kg). The hair between the two ears was removed, the skin was cut, and the subcutaneous tissue was separated after disinfection. The rats then received stereotaxic injection of 6-hydroxydopamine (6-OHDA; Sigma, USA, 4 μg/μL in 0.02% ascorbic acid saline solution) into the left medial forebrain bundle (4.0 μL; AP 2.10 mm, ML 2.16 mm, DV 8.4–8.65 mm relative to bregma) via a 5 μL injection syringe ((Hamilton Company, Reno, NV, USA) at a rate of 1 μL/min to degenerate dopaminergic neurons [28]. Sham animals received an equal volume of saline. Two weeks after lesioning, dopaminergic denervation was assessed using apomorphine (≥99%, Aladdin, Shanghai, China, 0.75 mg/kg, subcutaneous injection). After a 5 min habituation period, contralateral rotations were recorded for 15 min. Rats exhibiting more than 7 rotations per minute were considered successfully lesioned [29].

In the APO-induced rotation test, a total of 15 rats in the PD group and 15 in the LID group were confirmed to have a rotation rate exceeding 7 r/min, which is the criterion for successful model establishment. The 15 rats received chronic co-administration of levodopa (L-DOPA, ≥99%, MEC, Amagasaki, Japan, 2 mg/mL) and benserazide (≥99%, MEC, Japan, 3 mg/mL), whereas PD rats received saline injections.

### 2.3. LID Induction and Behavioural Assessment

Beginning on postoperative day 28, rats in the LID group (n = 15) were administered intraperitoneal injections of L-DOPA (8 mg/kg) and benserazide (12 mg/kg) every other day to establish the LID model. Abnormal involuntary movements (AIMs) were assessed after each injection using established criteria [30]. Rats were placed individually in a recording cage and observed across ten 5 min sessions, which were conducted every 20 min, for a total observation time of 185 min. During the first 2 min of each observation session, limb, axial, orolingual, and locomotor dyskinesia were scored independently using a 0–4 rating scale, with higher scores indicating greater dyskinesia severity [9].

### 2.4. Electrode Implantation

On day 21 after 6-OHDA lesioning, all rats were anesthetized with sodium pentobarbital (Sigma, USA, 30 mg/kg), and electrode arrays were implanted into the ipsilateral DLS and M1. Each array consisted of 16 Teflon-insulated nickel–chromium microwires with an exposed tip of approximately 2 mm. Stereotaxic coordinates were as follows: DLS: AP 0.0 mm, ML 3.5 mm, DV −5.0 mm; M1: AP 2.0 mm, ML 2.0 mm, DV −1.8 mm [28]. Three stainless steel screws were implanted on the surface of the rat skull to wind and fix the ground wire. The electrodes were fixed to the skull surface using dental acrylic and screws. After the operation, penicillin and meloxicam were injected intramuscularly for three consecutive days to prevent infection and relieve pain. Sham animals received equal operations.

### 2.5. Histological Verification

After completing the recordings, rats were deeply anesthetized with sodium pentobarbital (Sigma, USA, 200 mg/kg) and transcardially perfused. Brains were then removed and coronally sectioned at 5 μm thickness. Nissl staining was conducted to verify the electrode placement in the DLS and M1.

To assess dopaminergic neuron survival, immunohistochemical staining for tyrosine hydroxylase (TH) was performed [31]. Sections were incubated with a mouse anti-TH primary antibody (TH, 1:1000, HK25859-AP, Biotechnology Co, Ltd., Hangzhou, China), followed by biotinylated secondary antibodies (POLY-HRP Goat anti-rabbit IgG, HKI0026, Biotechnology Co, Ltd., China) and visualization using a DAB (Proteintech, Rosemont, IL, USA) detection system. TH-positive neurons in the substantia nigra pars compacta (SNc) were quantified.

### 2.6. Electrophysiological Recordings

Neural signals were recorded using a 256-channel data acquisition system (Blackrock Microsystems, Salt Lake City, UT, USA) at a sampling rate of 30 kHz. Recordings were performed in awake, freely moving rats simultaneously with behavioral assessments. Electrophysiological data were collected at 20 min intervals for 185 min following L-DOPA administration. Based on the time course of dyskinesia observed in the present study and previous reports, the 5 min recording obtained 85 min after L-DOPA administration, corresponding to the peak dyskinesia period, was selected for subsequent analyses [9].

### 2.7. LFP Analysis

Raw signals were notch-filtered at 50, 150, and 250 Hz to remove line noise and harmonics, followed by low-pass filtering at 270 Hz using a fourth-order zero-phase Butterworth filter. Signals were then downsampled to 1 kHz. Power spectral density (PSD) was estimated using Welch’s method with 2 s Hamming windows and 50% overlap. Relative power was calculated for standard frequency bands: delta (0.1–4 Hz), theta (4–8 Hz), alpha (8–12 Hz), beta (12–30 Hz), low-gamma (30–60 Hz), and high-gamma (60–120 Hz), normalized to total power. For group-level analysis, PSD values were averaged across channels within each animal and then across animals within each group.

### 2.8. Spike Sorting and Classification

Spike signals were band-pass filtered between 300 and 4000 Hz. Events exceeding a negative threshold of 5.5 standard deviations were detected, with a refractory period of 20 samples. Waveforms (10 pre-threshold and 37 post-threshold samples) were extracted and sorted using Offline Sorter (Plexon, Dallas, TX, USA) and MATLAB2024a. Principal component analysis was used for feature extraction, followed by k-means clustering. Neurons were classified into subtypes based on waveform shape, duration, and firing patterns, yielding three classes in both DLS and M1. Firing rates were calculated as spike counts divided by recording duration.

### 2.9. Phase-Amplitude Coupling (PAC) Analysis

Phase–amplitude coupling (PAC) was quantified using a modulation index (MI)-based method implemented in MATLAB. Low-frequency phase (1–12 Hz) and high-frequency amplitude (30–200 Hz) were extracted using band-pass filtering followed by the Hilbert transform. For intra-regional PAC, both phase and amplitude were derived from the same brain region, whereas for cross-regional PAC, the phase signal from one region was paired with the amplitude signal from the other region. Comodulograms were constructed using 18 phase bins. Statistical significance was assessed using a surrogate analysis approach, in which the amplitude time series was circularly shifted relative to the phase time series to generate a null distribution. The 95th percentile of the surrogate distribution was used as the significance threshold.

To ensure that dominant PAC estimates were not driven by isolated noise-related maxima, we further assessed the within-recording stability of PAC peaks. Each 5 min recording was divided into consecutive non-overlapping 10 s artifact-free epochs, and PAC comodulograms were computed independently for each epoch using the same frequency ranges and the same surrogate-based thresholding procedure. A recording was considered to contain a stable coupling peak only if a suprathreshold PAC cluster was reproducibly observed across all epochs and showed a consistent location in both the phase-frequency and amplitude-frequency dimensions. This criterion was applied uniformly to all experimental groups, and was independent of the expected frequency band, disease condition, or direction of cross-regional coupling.

To quantify dominant coupling, centroid-based measures—including centroid MI, phase frequency, and amplitude frequency—were calculated from the top 10% of suprathreshold PAC values within recordings that met the stability criterion. Recordings that did not exhibit a reproducible suprathreshold PAC structure were excluded from centroid-based PAC analyses. Statistical comparisons were performed only on summary PAC metrics (MI and centroid value), rather than individual frequency bins. Therefore, correction for multiple comparisons across frequency bins was not required. The numbers of included and excluded recordings for PAC analyses in each group are reported in Appendix A Table A1.

### 2.10. Statistical Analysis

The primary analyses of this study focused on PAC metrics in the DLS and M1, whereas LFP spectral power, neuronal firing rates, and AIM scores were analyzed as complementary outcome measures. Statistical analyses were subsequently performed as described below.

All data are presented as mean ± standard error of the mean (SEM). Statistical analyses were performed using GraphPad Prism 10.1.2. Normality was assessed using the Shapiro–Wilk test, and homogeneity of variances was evaluated using Welch’s test. For two-group comparisons, normally distributed data were analyzed using Welch’s unpaired *t*-test, whereas non-normally distributed data were analyzed using the Mann–Whitney U test. For comparisons involving three or more groups, Welch’s one-way ANOVA was applied for normally distributed data, followed by Dunn’s multiple comparisons test where appropriate, while the Kruskal–Wallis test was used for non-normally distributed data. All statistical analyses were performed using the individual animal as the statistical unit. Statistical significance was set at *p* < 0.05.

No formal sample size calculation was performed before the study. Sample sizes were determined based on previous electrophysiological studies using comparable animal models and experimental paradigms.

## 3. Results

### 3.1. Behavioural Validation of PD and LID Models

**Figure 1:**
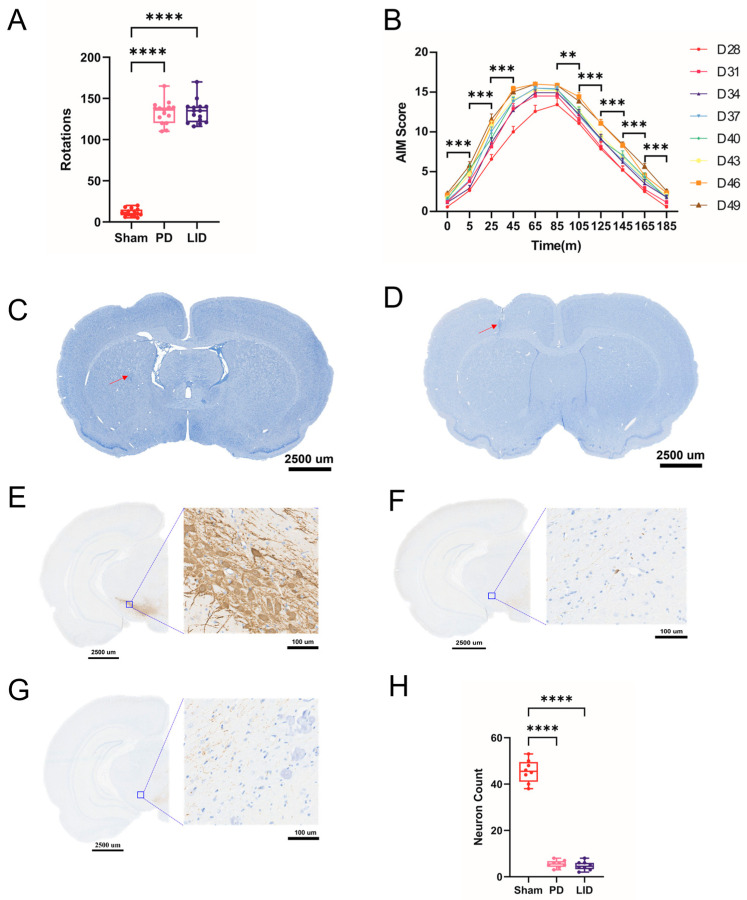
Behavioral validation of the PD and LID models. (**A**) Quantification of APO-induced rotations across groups (sham: N = 15; PD: N = 15; LID: N = 15). (**B**) Time course of AIM scores from before to after-185 min following L-DOPA administration during days 28–49(N = 14). (**C**,**D**) Representative electrode implantation sites in the M1 (**C**) and DLS (**D**). Red arrows indicate the electrode tips. (**E**–**G**) Representative images of TH-positive neurons in the SNc in sham (**E**), PD (**F**), and LID (**G**) rats. (**H**) Quantification of TH-positive neurons in the SNc across groups (sham: N = 8; PD: N = 8; LID: N = 8). Data are presented as mean ± SEM, where N indicates the number of animals included for statistical analysis. ** *p* < 0.01, *** *p* < 0.001, **** *p* < 0.0001 (Welch’s ANOVA test followed by Dunn’s multiple comparison test).

Behavioural assessments confirmed the successful establishment of PD and LID models. In the apomorphine-induced rotation test, Welch’s ANOVA results showed a significant difference among the three groups. Compared with the sham group, both PD and LID rats exhibited markedly increased contralateral rotations, as shown in Figure 1A (Welch’s ANOVA, W = 910.9 (2.000, 21.84), *p* < 0.0001), indicating robust unilateral motor dysfunction.

To further evaluate dyskinetic phenotypes, abnormal involuntary movements (AIMs) were quantified following repeated L-DOPA administration. As shown in Figure 1B, dyskinetic behaviours emerged rapidly within 5 min post-injection, progressively increased, and reached a peak between 65 and 85 min, followed by a gradual decline (Welch’s ANOVA, W = 252.3 (10.000, 30.62), *p* < 0.0001). Repeated-measures analysis across recording days showed no significant variation in AIM scores, indicating stable expression of dyskinesia over time. These behavioural results validate both the reliability of the lesion model and the successful induction of LID.

### 3.2. Histological Verification of Lesions and Electrode Placement

Histological analyses confirmed accurate electrode placement and successful dopaminergic lesions. Nissl staining verified the localization of electrode tracks within the dorsolateral striatum (DLS) and primary motor cortex (M1), with no apparent tissue damage beyond the implantation sites (Figure 1C,D). Animals with incorrect electrode placement were excluded. Accordingly, a total of 7 control, 8 PD, and 8 LID rats were included in the final analyses. Detailed exclusion information is provided in Appendix A Table A1.

Immunohistochemical staining of tyrosine hydroxylase (TH) revealed a marked loss of dopaminergic neurons in the ipsilesional substantia nigra pars compacta (SNc) in both PD (Figure 1F) and LID (Figure 1G) rats compared with sham (Figure 1E) (Welch’s ANOVA, W = 237.2 (2.000, 12.78), *p* < 0.0001) (Figure 1H), with no significant difference between the PD and LID groups. These findings confirm that the observed electrophysiological changes are not due to differences in lesion severity, but rather reflect functional alterations induced by L-DOPA exposure.

### 3.3. LFP Alterations in DLS

**Figure 2:**
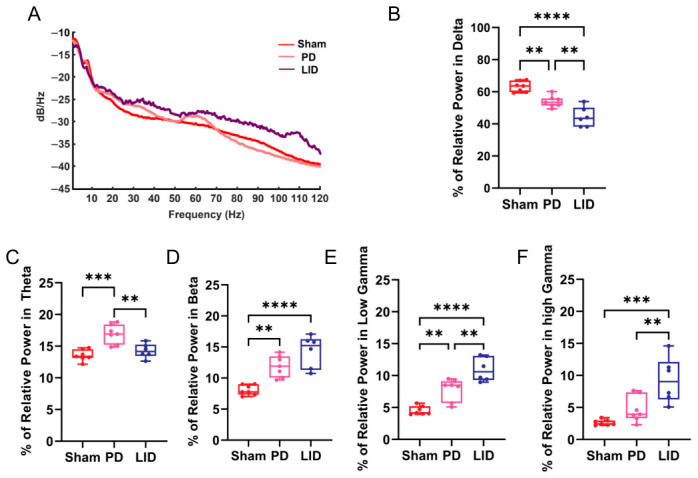
PSD curves and band-specific oscillatory activity in the DLS. (**A**) PSD plots in the DLS. (**B**–**F**) Quantification of relative power in different frequency bands: delta (**B**), theta (**C**), beta (**D**), low gamma (**E**), and high gamma (**F**). The LID group showed decreased delta power, whereas beta and gamma oscillations were significantly increased in both the PD and LID groups. Data are expressed as mean ± SEM (sham: N = 7; PD: N = 7; LID: N = 6), where N indicates the number of animals included for statistical analysis. ** *p* < 0.01, *** *p* < 0.001, **** *p* < 0.0001 (Welch’s ANOVA test followed by Dunn’s multiple comparison test).

LFP analysis revealed pronounced frequency-specific alterations in striatal oscillatory activity across groups (Welch’s ANOVA). Compared with the sham group, both PD and LID rats exhibited a significant reduction in the delta band (0.1–4 Hz) (W = 25.91(2.000, 10.25), *p* < 0.0001, Figure 2B), with a further decrease observed in LID relative to PD. Theta band activity (4–8 Hz) (Welch’s ANOVA, W = 12.58 (2.000, 10.63), *p* = 0.0016) was increased in PD rats but significantly reduced in LID rats (Figure 2C), indicating that chronic L-DOPA treatment reverses PD-associated theta enhancement. In contrast, beta (12–30 Hz) power was significantly elevated in both the PD and LID groups compared with sham (Welch’s ANOVA, W = 25.23 (2.000, 8.936), *p* = 0.0002) (Figure 2D), with no significant difference between the PD and LID groups. This suggests that beta abnormalities are primarily associated with dopamine depletion rather than dyskinesia. Notably, both low-gamma (30–60 Hz) (Welch’s ANOVA, W = 36.34 (2.000, 8.845), *p* < 0.0001) (Figure 2E) and high-gamma power (60–120 Hz) (Welch’s ANOVA, W = 12.42 (2.000, 7.659), *p* = 0.0039) (Figure 2F) exhibited significant increases in the PD and LID groups, with a further enhancement in LID. Overall, these results suggest a shift in DLS oscillatory dynamics from low-frequency dominance toward enhanced high-frequency activity under dyskinetic conditions.

### 3.4. Spike Activity in DLS

**Figure 3:**
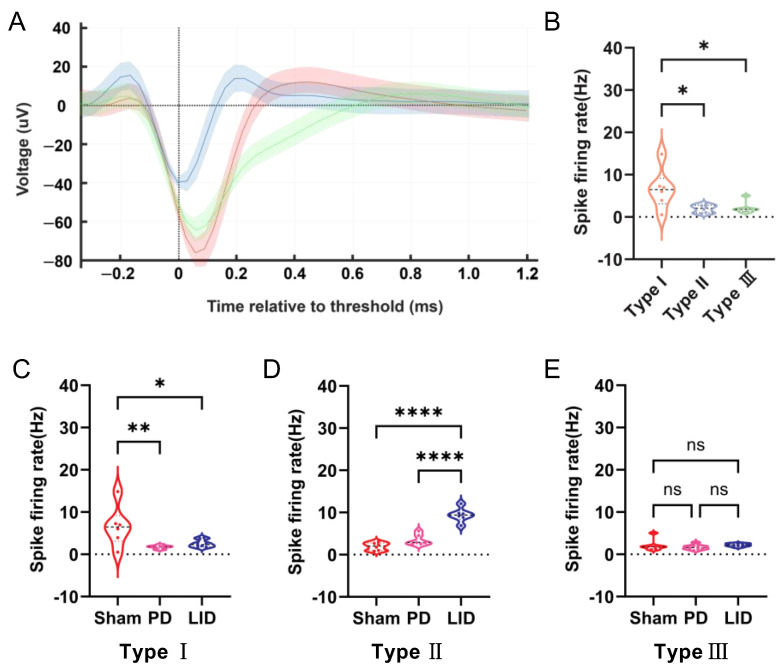
Spike classification and firing rate analysis of neurons in the DLS. (**A**) Representative waveforms of three neuronal types (Type I–III). Solid lines indicate averaged waveforms. (**B**) Comparison of firing rates among neuronal types under the sham condition. (**C**–**E**) Firing rates of Type I (**C**), Type II (**D**), and Type III (**E**) neurons across groups. Alterations in firing rates were observed in specific neuronal subtypes across experimental conditions. Data are presented as mean ± SEM (sham: N = 7; PD: N = 8; LID: N = 8), where N indicates the number of animals included for statistical analysis (number of neurons per group indicated in the main text). * *p* < 0.05, ** *p* < 0.01, **** *p* < 0.0001 (Welch’s ANOVA and Kruskal–Wallis test followed by Dunn’s multiple comparison test).

Spike sorting identified three distinct neuronal populations from a total of 583 neurons in the DLS based on waveform characteristics and firing patterns, as shown in Figure 3A. Type I neurons (n = 86, red line, 6 from sham, 62 from PD, 18 from LID) exhibited short-duration waveforms and high, irregular firing rates, consistent with putative fast-spiking interneurons (FSIs) [32]. Types II neurons (n = 163, blue line, 56 from sham, 87 from PD, 20 from LID) and Types III neurons (n = 334, green line, 115 from sham, 130 from PD, 89 from LID) displayed longer waveform durations and lower firing rates, consistent with putative medium spiny neurons (MSNs) [32,33,34]. Under sham conditions, no significant differences in firing rates were observed among the three neuronal types (Kruskal–Wallis test, H (3) = 4.810, *p* = 0.0875). Statistical comparisons of firing rates across groups were performed using per-animal averages.

Neuronal firing rates showed differential changes across experimental groups. Dunn’s multiple comparisons test revealed that type I neurons exhibited significantly reduced firing rates in both the PD and LID groups compared with the sham group (Welch’s ANOVA, W (2.000, 8.919) =5.395, *p* = 0.0291) (Figure 3C). In contrast, type II neurons showed significantly increased firing rates in both the PD and LID groups, with a further increase in LID compared with PD (Welch’s ANOVA, W (2.000, 9.447) = 42.15, *p* < 0.0001) (Figure 3D), indicating enhanced excitatory output of projection neurons under dyskinetic conditions. No significant changes were observed for type III neurons (Kruskal–Wallis test, H (3) = 4.178, *p* = 0.1238) (Figure 3E). Overall, these results indicate altered striatal neuronal activity characterized by reduced type I neuron firing and increased type II neuron firing under PD and LID conditions. Statistical comparisons of firing rates were performed using per-animal averaged values.

### 3.5. PAC Alterations in DLS

**Figure 4:**
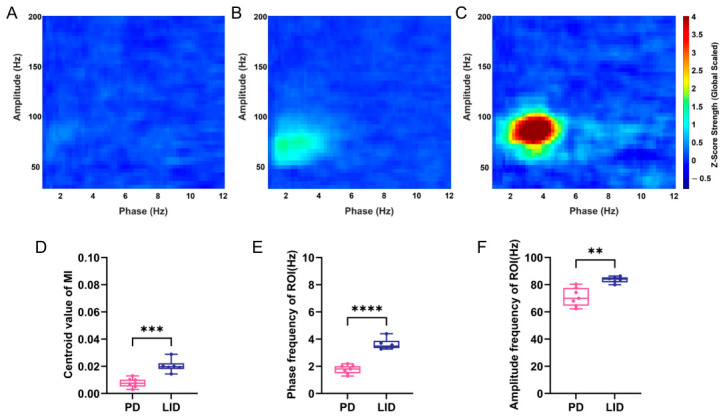
PAC in the DLS. (**A**–**C**) Representative PAC comodulograms in the sham (**A**), PD (**B**), and LID (**C**) groups. (**D**) Modulation index (MI) at the centroid. (**E**) Phase frequency at the centroid. (**F**) Amplitude frequency at the centroid. PAC characteristics differed between the PD and LID groups in both coupling strength and frequency distribution. Data are presented as mean ± SEM (PD: N = 8; LID: N = 7), where N indicates the number of animals included for statistical analysis. ** *p* < 0.01, *** *p* < 0.001, **** *p* < 0.0001 (Welch’s *t*-test).

Phase-amplitude coupling analysis revealed alterations in DLS oscillatory coupling under pathological conditions. In sham rats, low levels of PAC were observed (Figure 4A), indicating weak phase–amplitude coupling under physiological conditions. In contrast, both PD and LID groups exhibited increased coupling between low-frequency phase and high-frequency amplitude, with more pronounced effects in the LID group (Figure 4B,C). Welch’s Student’s *t*-test showed that the modulation index (MI) was significantly increased in LID compared with PD (t (8.913) = 5.338, *p* = 0.0005) (Figure 4D). In addition, the preferred phase frequency shifted toward higher values (t (9.377) = 8.869, *p* < 0.0001) (Figure 4E), and the coupled amplitude frequency also differed between groups (t (7.552) = 4.682, *p* = 0.0018) (Figure 4F). Overall, these results indicate altered DLS PAC profiles in LID, characterized by increased coupling strength and a shift in dominant frequency components.

### 3.6. LFP Alterations in M1

**Figure 5:**
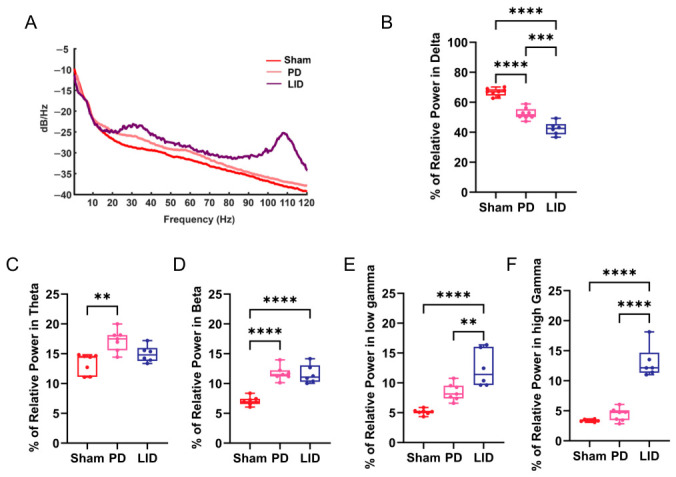
PSD and oscillatory activity in the M1. (**A**) PSD plot in the M1. (**B**–**F**) Relative power in delta (**B**), theta (**C**), beta (**D**), low gamma (**E**), and high gamma (**F**) bands. Frequency-dependent differences in oscillatory activity were observed across groups in the M1. Data are presented as mean ± SEM (sham: N = 7; PD: N = 7; LID: N = 6), where N indicates the number of animals included for statistical analysis. ** *p* < 0.01, *** *p* < 0.001, **** *p* < 0.0001 (Kruskal–Wallis and Welch’s ANOVA test followed by Dunn’s multiple comparison test).

Oscillatory activity in M1 showed patterns broadly similar to those observed in the DLS, with region-specific differences, as illustrated by the PSD results in Figure 5A. Compared with both sham and PD groups, LID rats exhibited a significant reduction in delta-band power (Welch’s ANOVA, W (2.000, 9.778) = 101.3, *p* < 0.0001) (Figure 5B). Theta-band (Kruskal–Wallis test, H (3) = 9.223, *p* = 0.0052) and beta-band (Welch’s ANOVA, W (2.000, 9.786) = 46.39, *p* < 0.0001) power were increased in the PD group relative to the sham group, but did not differ significantly between the PD and LID groups (Figure 5C,D). In contrast, both low-gamma (Welch’s ANOVA, W (2.000, 8.032) = 29.43, *p* = 0.0002) and high-gamma power (Welch’s ANOVA, W (2.000, 7.506) = 39.68, *p* = 0.0001) were significantly higher in the LID group compared with the PD group, while no differences were observed between the PD and sham groups (Figure 5E,F). Overall, these results indicate that L-DOPA treatment is associated with increased high-frequency oscillatory activity in the cortex under dyskinetic conditions.

### 3.7. Spike Activity in M1

**Figure 6:**
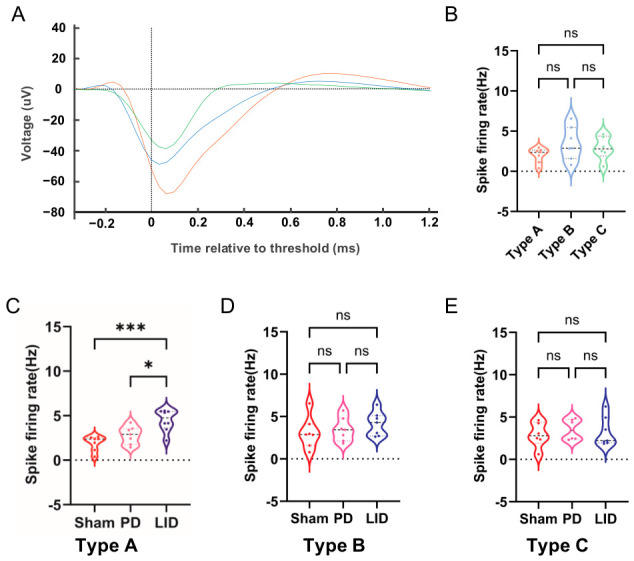
Spike activity and neuronal firing properties of neurons in M1. (**A**) Representative waveforms of Type A–C neurons. (**B**) Comparison of firing rates among neuronal types under the sham condition. (**C**–**E**) Firing rates of Type A (**C**), Type B (**D**), and Type C (**E**) neurons across groups. Subtype-specific differences in neuronal firing activity were observed under different experimental conditions. Data are presented as mean ± SEM. (sham: N = 7; PD: N = 8; LID: N = 8), where N indicates the number of animals included for statistical analysis (the number of neurons per group is indicated in the main text). * *p* < 0.05, *** *p* < 0.001 (Kruskal–Wallis and Welch’s ANOVA test followed by Dunn’s multiple comparison test). The above statistical analysis was performed at the animal level.

Spike analysis in the M1 identified three neuronal subtypes from a total of 753 neurons based on waveform characteristics and firing patterns (Figure 6A). Type A neurons (n = 313, red line, 107 from sham, 133 from PD, 73 from LID) and Type B neurons (n = 346, blue line, 136 from sham, 144 from PD, 66 from LID), both characterized by broader waveforms, were classified as putative pyramidal neurons. In contrast, Type C neurons (n = 94, green line, 21 from sham, 19 from PD, 54 from LID), characterized by narrower waveforms, were classified as putative interneurons [35]. Under sham conditions, no significant differences in firing rates were observed among the three neuronal types (Welch’s ANOVA, W (2.000, 9.943) = 1.825, *p* = 0.2113) (Figure 6B), followed by post hoc Dunn’s multiple comparisons test.

Alterations in both neuronal composition and firing activity were observed in LID rats. The number of pyramidal neurons (Type A, n = 73 and Type B, n = 66) was reduced in the LID group compared with the sham group (Type A, n = 107, Type B, n = 136) and PD (Type A, n = 133, Type B, n = 144), whereas the number of Type C neurons was increased (sham, n = 21, PD, n = 19, LID, n = 54). Regarding firing activity, Type A neurons exhibited significantly increased firing rates in the LID group (Welch’s ANOVA, W (2.000, 12.58) = 10.90, *p* = 0.0018) (Figure 6C), whereas no significant differences were observed in Type B neurons (Welch’s ANOVA, W (2.000, 11.83) = 0.7461, *p* = 0.4952) or Type C neurons (Kruskal–Wallis test, H(3) =0.7947, *p* = 0.6860) (Figure 6D,E). Overall, these results indicate altered neuronal composition and firing patterns in the M1 under LID conditions. All statistical analyses were performed using per-animal values as independent biological replicates.

### 3.8. PAC in M1

**Figure A1:**
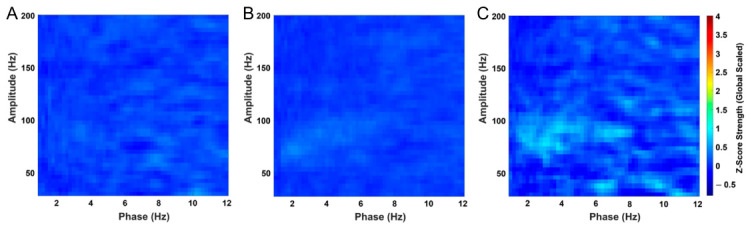
Effects of L-DOPA on PAC in M1. (**A**–**C**) Representative PAC comodulograms in sham (**A**), PD (**B**), and LID (**C**) groups.

In contrast to the results obtained from the DLS, no significant PAC patterns were detected in M1 across all groups (Appendix A Figure A1). The comodulogram analysis did not reveal coupling that exceeded surrogate thresholds, indicating weak or non-significant phase–amplitude interactions in M1 under both physiological and pathological conditions.

### 3.9. Cross-Regional PAC Between M1 and DLS

**Figure 7:**
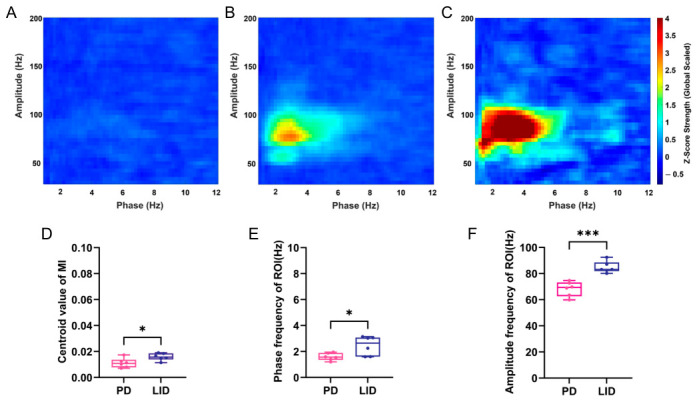
Cross-regional PAC from M1 phase to DLS amplitude. (**A**–**C**) Representative PAC comodulograms showing modulation of DLS amplitude by M1 phase in sham (**A**), PD (**B**), and LID (**C**) groups. (**D**) Modulation index (MI) at the centroid. (**E**) Phase frequency at the centroid. (**F**) Amplitude frequency at the centroid. Cross-regional PAC differed between PD and LID groups. Data are presented as mean ± SEM (PD: n = 6; LID: n = 6), where N indicates the number of animals included for statistical analysis. * *p* < 0.05, *** *p* < 0.001 (Welch’s *t*-test).

Under the LID condition, cross-regional PAC analysis revealed stronger coupling between the low-frequency phase in the M1 and the high-frequency amplitude in the DLS compared with the reverse pairing (DLS phase-M1 amplitude). In particular, coupling involving the delta-band phase in M1 and gamma-band amplitude in DLS was observed. Compared with the PD group, the LID group exhibited a broader distribution and higher magnitude of coupling (Figure 7A–C). Welch’s *t*-test showed a significant increase in modulation index (MI) in the LID group (t (9.325) = 2.537, *p* = 0.0310) (Figure 7D). Furthermore, the preferred phase frequency shifted toward broader frequencies (t (6.459) = 2.666, *p* = 0.0347), while the amplitude frequency increased within the high-gamma range (t (9.492) = 5.676, *p* = 0.0002) (Figure 7E–F). Overall, these results indicate altered cross-regional coupling patterns within the cortico-basal ganglia network under LID conditions.

**Figure A2:**
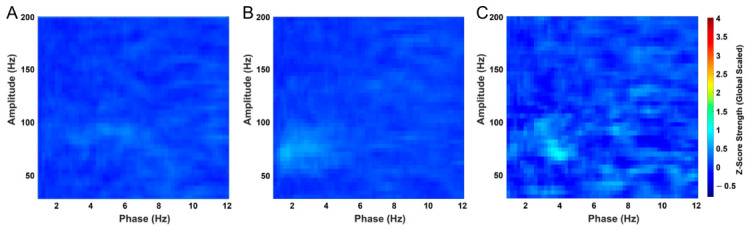
Effects of L-DOPA on PAC in DLS phase to M1 amplitude. (**A**–**C**) Representative PAC comodulograms showing modulation of M1 amplitude by DLS phase in sham (**A**), PD (**B**), and LID (**C**) groups.

In contrast, no significant coupling was detected in the reverse pairing (Appendix A Figure A2). These findings suggest asymmetric cross-regional PAC patterns in LID.

**Figure A3:**
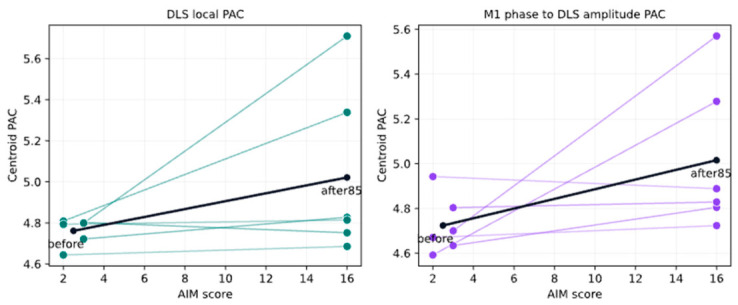
Descriptive AIM-PAC trajectories in selected LID rats. Animal-level AIM-PAC trajectories from before L-DOPA administration to the after-85 time point are shown for DLS local PAC and M1 phase-to-DLS amplitude PAC. Each thin line represents one rat, and the thick black line indicates the group mean trajectory. The y-axis shows the PAC centroid value, consistent with the PAC metric used in the main analysis, and the x-axis shows the AIM score. Most selected animals showed an increase in PAC centroid values as AIM scores increased. However, the absolute PAC strength exhibited notable inter-animal variability; therefore, this analysis should be interpreted as descriptive and hypothesis-generating.

To further explore the relationship between behavioral measures and PAC alterations, we performed a descriptive animal-level analysis in selected LID rats with paired AIM and PAC measurements obtained before L-DOPA administration and at the 85 min time point. As shown in Appendix A Figure A3, individual animals displayed concurrent changes in AIM scores and PAC centroid values, particularly in M1 phase-to-DLS amplitude coupling and local DLS PAC measures. Given the substantial inter-animal variability in absolute PAC values, this analysis was presented as a descriptive supplementary observation rather than a formal correlation analysis between AIM and PAC metrics.

## 4. Discussion

In the present study, we characterized electrophysiological alterations across multiple spatial and temporal scales within the cortico-striatal circuit in LID rats. By integrating single-unit activity, LFPs, and both intra- and inter-regional PAC, we identified three main features associated with LID: (1) changes in oscillatory activity characterized by reduced delta and enhanced gamma power; (2) cell-type-specific alterations in neuronal firing patterns in both the DLS and M1; (3) an asymmetric pattern of M1-phase to DLS-amplitude coupling. Overall, these findings suggest that LID involves coordinated alterations in local circuit activity and cross-regional coupling within the cortico-striatal network.

### 4.1. Oscillatory Reorganization Reflects a Shift Toward Hyperexcitable Network States in LID

Our results show a consistent decrease in delta-band power accompanied by increased gamma oscillations in both the DLS and M1 under dyskinetic conditions. Delta oscillations are typically associated with large-scale synchronization and low-excitability network states [36], whereas gamma activity reflects local circuit excitation and active information processing [16,37,38]. The observed reduction in delta power, together with enhanced gamma activity, suggests a shift from coordinated slow rhythms to hyperactive and desynchronized network dynamics in LID. This pattern is consistent with previous studies reporting that elevated gamma oscillations are a characteristic feature of dyskinetic states and are associated with abnormal motor output [16,39]. At a mechanistic level, increased gamma activity may reflect altered balance between excitatory and inhibitory network activity, while reduced delta power may be associated with impaired large-scale temporal coordination, potentially affecting the interaction between local and global neural dynamics.

Theta oscillations showed divergent changes between PD and LID, increasing in PD but decreasing in LID. These differences suggest condition-dependent modulation of theta-band activity [5,11,12,40,41]. Overall, LID is associated with frequency-specific changes in spectral activity across multiple bands.

### 4.2. Microcircuit Remodeling Disrupts Excitation–Inhibition Balance

At the level of single neurons, distinct yet complementary alterations were observed across neuronal subtypes in both the DLS and M1, highlighting the importance of cell-type-specific mechanisms in LID. In the DLS, putative FSIs exhibited reduced firing rates but increased representation [33], whereas putative MSNs showed elevated firing activity. Given the strong inhibitory control that FSIs exert over MSNs [32,34], reduced activity of putative FSIs may contribute to altered inhibitory control over MSNs [42]. This imbalance is consistent with changes in basal ganglia circuit function associated with dyskinesia [43]. Previous studies have reported that chronic L-DOPA treatment is associated with increased excitability of direct pathway MSNs and abnormal motor output [32,34,42]. In line with these observations, our results show increased firing of putative MSNs together with reduced putative FSI activity. In addition, the increased proportion of putative FSIs may represent a compensatory change in response to network activity alterations, although this potential adaptation does not appear sufficient to fully restore inhibitory balance.

In M1, putative pyramidal neurons showed increased firing rates despite a reduced proportion, whereas putative interneurons were more abundant and exhibited no significant change in firing rate [35,44]. This pattern suggests altered cortical neuronal activity characterized by changes in the balance between excitatory and inhibitory elements. These changes may be associated with altered synaptic and network inputs under chronic L-DOPA treatment [45]. Collectively, these findings indicate that LID involves coordinated alterations in neuronal activity across both striatal and cortical regions, which may contribute to the observed oscillatory abnormalities.

### 4.3. Enhanced Striatal PAC Reflects Aberrant Local Integration of Neural Signals

PAC analysis revealed regional differences in network organization. In the DLS, enhanced coupling was observed between low-frequency (delta/theta) phase and high-frequency gamma amplitude in LID rats, together with a shift toward higher gamma frequency components. This pattern suggests increased modulation of high-frequency activity by low-frequency oscillations, which may be associated with altered temporal structuring of neuronal activity. Under physiological conditions, PAC is considered an electrophysiological feature of coordinated neuronal activity across multiple temporal scales and may reflect the temporal organization of neural dynamics within and across brain regions. In contrast, abnormal PAC patterns may be associated with altered synchronization of neuronal populations, potentially reflecting changes in network coordination [46,47]. In the context of LID, enhanced delta–gamma coupling in the striatum may be associated with a more constrained oscillatory dynamic state within local circuits, potentially reflecting reduced flexibility in the regulation of neuronal activity [18,38,46]. The observed shift toward higher gamma frequencies may reflect altered excitability-related dynamics within striatal circuits. This change could be associated with dopaminergic modulation of synaptic and network activity, potentially contributing to the emergence of abnormal oscillatory patterns. Overall, these findings suggest that the striatum may play an important role in the integration of altered neural signals in LID.

### 4.4. Absence of Cortical PAC Suggests Dependence on External Inputs

In contrast to the DLS, no significant enhancement of PAC was detected in the M1 region. This regional difference suggests that LID-related alterations in cross-frequency coupling are not uniformly distributed across the motor network, but may be more prominent within striatal circuits. As the primary target of dopaminergic replacement therapy, the striatum is known to undergo substantial changes in neuronal excitability and synaptic plasticity in LID. Together with the observed alterations in the firing properties of putative MSNs and FSIs, these findings suggest that dopaminergic modulation may contribute to an imbalance between excitatory and inhibitory activity within striatal microcircuits, which may be associated with altered coordination of neuronal population activity and changes in gamma-band modulation by low-frequency oscillations. Accordingly, the enhanced PAC observed in the DLS may reflect altered local network dynamics related to motor information processing within the striatum.

In contrast, although oscillatory power changes were also observed in M1, these were not accompanied by significant alterations in local PAC. This suggests that cortical oscillatory abnormalities may primarily reflect changes in overall neuronal activity or interregional interactions rather than reorganization of local cross-frequency coupling. Given the complex cytoarchitecture and heterogeneous inputs of M1, neuronal activity in this region is likely influenced by multiple converging signals, which may contribute to the absence of stable local PAC changes. Therefore, the role of M1 in LID may be more related to the integration and transmission of altered motor-related signals, whereas more pronounced changes in cross-frequency coupling appear to be more evident within striatal circuits. Overall, these findings are consistent with a prominent involvement of striatal microcircuit alterations in L-DOPA-induced dyskinesia.

### 4.5. Asymmetric Enhancement of PAC Is Associated with Altered Cortico-Striatal Coordination During LID

A key observation of this study is an asymmetric pattern of cross-regional phase–amplitude coupling (PAC) between M1 and DLS in LID. Specifically, coupling between the low-frequency delta phase in M1 and high-frequency gamma amplitude in the DLS was observed, whereas no significant coupling was detected in the reverse pairing. This asymmetry suggests the presence of direction-dependent differences in cross-regional PAC within the cortico-striatal circuit [7]. Under LID conditions, enhanced M1-phase to DLS-amplitude coupling was observed, indicating an asymmetric pattern of cross-regional phase–amplitude coupling between the cortex and the striatum. Several anatomical and functional characteristics of the cortico-striatal system may be relevant to this observation. First, cortico-striatal projections from M1 to DLS are anatomically prominent, whereas feedback pathways are less direct [48,49,50]. Second, we speculate that pulsatile dopaminergic stimulation may exert more direct modulation on striatal dopaminergic neurons, while its modulation of M1 is mediated through feedback pathways, which may explain why we did not observe symmetrical changes in PAC between DLS and M1 [43,51]. Therefore, this asymmetry likely reflects the inherent structural and physiological constraints of the circuitry itself. The observed shift toward lower phase frequencies may reflect changes in the contribution of slow cortical oscillations to cross-regional interactions, while the increase in gamma amplitude frequency may be associated with enhanced local high-frequency activity [48,49,50,52]. Notably, this direction-dependent PAC pattern suggests that LID may be associated with asymmetric cross-regional coupling, alongside alterations in local oscillatory activity.

Nevertheless, it should be noted that PAC reflects asymmetric patterns of cross-regional cross-frequency coupling, but does not, on its own, establish causal relationships underlying the observed abnormalities. To address this limitation, future studies will further investigate both PAC and Granger causality between the DLS and M1 across the time course of L-DOPA treatment, which may help to better characterize directional interactions between these regions in the LID state.

### 4.6. Implications for Neuromodulation

The asymmetric coupling pattern may have potential value as a candidate biomarker for LID. Current neuromodulation approaches, such as deep brain stimulation (DBS), primarily target local oscillatory activity. However, the extent to which these approaches fully address network-level alterations underlying dyskinesia remains unclear. By capturing both frequency-specific dynamics and cross-regional coupling, PAC may provide a complementary measure of neural coordination. Modulation of this asymmetric coupling may represent a potential direction for further exploration of circuit-level interventions in pathological networks.

## 5. Conclusions

In summary, LID is characterized by coordinated alterations in oscillatory activity, neuronal firing, and cross-regional coupling within the cortico-striatal circuit. The emergence of asymmetric M1–DLS PAC suggests altered cortico-striatal coupling in dyskinesia and may provide a basis for further investigation of network-level mechanisms underlying LID. While this study addresses its primary objectives, several limitations should be acknowledged to guide future work. First, electrophysiological signals were analyzed only at the peak of dyskinesia (85 min post-injection), without capturing dynamic changes across the full time course. Second, recordings were limited to the DLS and M1, and did not include other key nodes such as the thalamus or globus pallidus. Future studies will further investigate the potential utility of M1–DLS PAC as a biomarker of network dynamics in LID and explore its relationship with disease progression and circuit-level alterations in animal models.

## 6. Limitations

Several limitations should be noted. First, blinding was not performed during AIM scoring, histological assessment, spike sorting, or data analysis, which may have introduced bias. Second, only male Sprague-Dawley rats were used, which limits the generalizability of our findings to females or other strains. Third, neuronal subtype classification is based on extracellular waveform characteristics and firing properties, and this approach lacks direct experimental validation. Finally, in accordance with the 3R principles (Replacement, Reduction, and Refinement) for animal research, the number of animals used was minimized while maintaining the scientific objectives of the study. Consequently, the relatively small sample size may have reduced the statistical power of the analyses. These factors should all be taken into consideration when interpreting the results.

## Data Availability

The data supporting the findings of this study are available from the corresponding author upon reasonable request, and the source code used for data analysis is also available from the corresponding author upon reasonable request. Access to the data and code may be subject to institutional regulations, ethical approvals, and reasonable restrictions related to ongoing research.

## Abbreviations

The following abbreviations are used in this manuscript:

ANOVA	One-way analysis of variance
AIMs	Abnormal involuntary movements
DBS	Deep brain stimulation
DLS	The dorsolateral striatum
FSIs	Fast-spiking interneurons
L-DOPA	Levodopa
LFP	Local field potentials
LID	Levodopa-induced dyskinesia
M1	The motor cortex
MI	Modulation index
MSNs	Medium spiny neurons
6-OHDA	6-hydroxydopamine
PAC	Phase amplitude coupling
PD	Parkinson’s disease
PSD	Power spectral density
SNc	Substantia nigra pars compacta
TH	Tyrosine hydroxylase

## Appendix A

**Table A1:** Summary of the Number of Animals Screened, Excluded, and Included in the Analysis.

Group	Initially Assigned	Successful Lesion/Sham	Implanted	Valid Histology (TH)	Valid Spike	Valid LFP	Valid PAC	Valid Cross-PAC
Sham	15	15 2 rats: died during the surgical procedure 1 rat: motor impairment in one limb after electrode implantation, rendering signal recording impossible.	12 2 rats: tissue damaged 2 rats: failed staining.	8 1 rat: No spike signal detected and full-channel noise	7	7	7	7
PD	15	15 1 rat: died in surgical mortality; 1 rat: electrode detachment occurred; 2 rats: incorrect electrode placement.	11 1 sample failed to be collected; 2 samples failed to stain.	8 1 rat: noise interference	8	7	7 1 rat: significant noise interference in the DLS region	6
LID	15 1 rat: LID induction failure	14 1 rat: electrode detachment occurred; 2 rats: incorrect electrode placement.	11 3 rats: electrode detachment occurred;	8 2 rats: motion noise and LFP were not analyzed.	8	6	6	6
